# Machine learning as the new approach to understand biomarkers of suicidal behavior

**DOI:** 10.17305/bjbms.2020.5146

**Published:** 2021-08

**Authors:** Alja Videtič Paska, Katarina Kouter

**Affiliations:** Medical Centre for Molecular Biology, Institute of Biochemistry, Faculty of Medicine, University of Ljubljana, Ljubljana, Slovenia

**Keywords:** Suicide, artificial intelligence, personalized medicine, precision medicine, precision psychiatry

## Abstract

Compared to other medical fields, the situation in psychiatry is particularly lacking in terms of identification of biological markers that can complement current clinical interviews. Such markers would enable more objective and rapid clinical diagnosis and allow more accurate monitoring of treatment responses and remission. Current technological developments can provide analyses of various biological marks at a high-throughput scale and at reasonable cost, and therefore such “-omic” studies are also now entering psychiatry research. However, big data demands a whole plethora of new skills in data processing before clinically useful information can be extracted. To date, the classical approaches to data analysis have not really contributed to identification of biomarkers in psychiatry. However, the extensive amount of data might be taken to a higher level if artificial intelligence can be applied, in the shape of machine learning algorithms. Not many studies on machine learning in psychiatry have been published, but we can already see from the handful of studies now available that the potential to build a screening portfolio of biomarkers for different psychopathologies, including suicide, exists.

## INTRODUCTION

One of the most important breakthroughs in biomedicine was the publication of the first draft of the human genome at the beginning of this millennium [1,2]. Since then, we have witnessed impressive technological developments, particularly with the birth of the many “-omic” approaches (e.g., genomics, transcriptomics, epigenomics, proteomics, metabolomics, and more). These have already made important ­contributions to the progress of classical medicine towards personalized medicine, and further on to “precision medicine.” The term “personalized medicine” indicates the development of a particular therapeutic path for each and every individual patient, based on their unique characteristics. These “unique characteristics” are very often influenced by the genetic background, and applications such as pharmacogenomics and gene therapies are thus used. Further advances, in particular in the field of genetics, are now the main drivers for development of what is called precision medicine –technology-driven and participant-centered approaches that can be used for disease classification [3,4].

For the case of antidepressant treatments, two recent meta-analyses have shown that important improvements can be obtained when treatments are guided using commercially available pharmacogenetic kits for genetic profiling (e.g., Neuropharmagen, GeneSight, CNSDose and NeuroIDgenetix). Indeed, the risk ratio for treatment responses between guided and nonguided treatments was 1.36 in favor of guided treatments [5], while symptom remission was 1.71-fold more likely to be achieved with guided treatments [6].

Every day, huge amounts of data in various forms are being collected and processed, from genomic to organ-imaging information. To bring these “-omic” data to the clinical level, tools for efficient data processing, analysis, and interpretation are being developed. However, with the complexities of the required actions increasing, the need for more and more powerful and intricate analysis and interpretation tools is also increasing. The major benefits that are expected to be gained by these sophisticated approaches are the identification, treatment, and monitoring of complex, multifactorial diseases. Here, the data on the disease symptoms that are accompanied by demographic and life-style information can be taken to a further level by the various molecular biology analyses where thousands of tiny bits of information are gathered.

Psychiatry is one of the fields that can particularly gain from such “-omics” data and new analytical approaches. Indeed, current psychiatric diagnosis is based on subjective clinical evaluation, without any molecular-genetics tests involved. In the era of personalized and precision medicine, mental disorders might be better explained, understood, and treated.

The aim of this review is to introduce machine learning and its most commonly used algorithms to showcase their potential use in psychiatry. Furthermore, we present practical examples of the use of machine learning with high-throughput data (i.e., genomics, metabolomics, epigenomics and imaging) associated with suicidal behavior. Finally, we discuss the challenges that need to be faced and the opportunities that machine learning can provide.

## MACHINE LEARNING AND PSYCHIATRY

### Machine learning

Compared to just a decade ago, the amount of data that is being produced has sky-rocketed. It is predicted that by the end of 2025, 175 zettabytes of data will have been produced [7]. In this era of big data, great computational power is needed to increase the dimensionality of the analyses and to help towards removing as much bias as possible.

When a computer is required to process a simple task, it is easy to give instructions by programing the computer. As the task complexity increases, it is often better if we allow the computer to identify the most efficient solutions without any further specific instructions in terms of how this is achieved, which takes us into the realms of machine learning.

**FIGURE 1:**
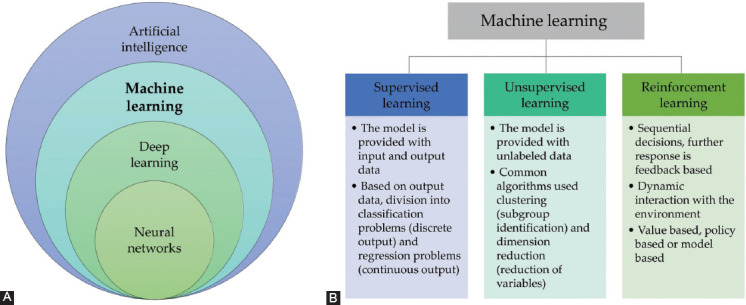
(A) Machine learning is an integral part of artificial intelligence. (B) Machine learning can be categorized into the three main fields of supervised learning, unsupervised learning, and reinforcement learning based on the purpose of the proposed model.

Machine learning is a relatively recent field that has developed from the discipline of artificial intelligence. A broadly used definition was given by Mitchell in 1997, when he stated “A computer program is said to learn from experience *E* with respect to some class of tasks *T* and performance measure *P*, if its performance at tasks in *T*, as measured by *P*, improves with experience *E*.” Machine learning is therefore based on the idea that a computer can learn from the data that it is given and can even improve its algorithms based on experience, without the need for further detailed instructions [8]. Machine learning can be further divided into the distinct categories of supervised learning, unsupervised learning, and reinforcement learning, which are based on the signals the model receives (Figure 1) [9].

### Supervised learning

The model is provided with labeled data, which means that the model already has the information as to which data are the input (feature or attribute; e.g., a person is a member of a psychiatric inpatient group or a member of a healthy control group; age), and which data are the output (outcome; e.g., presence of suicidal ideation). The job of the model is then to predict the outcome based on the association of previously observed input and output data. Often-used algorithms include regression, decision trees, and random forests [10]. Decision trees are a model based on hierarchy, which comprises the root, internal decision nodes, branches, and final leaf nodes. Each internal decision node (which represents a test) can provide multiple branches (which represent a test outcome) and lead to a new internal decision node or a final leaf node (the final outcome). The process starts at the root and ends at the final leaf node [10]. Random forests use a similar approach that creates and combines multiple decision trees. Contrary to the decision trees described above, each decision tree of a random forest is comprised of a random subset of the original dataset, and it can test random variables at nodes. The final outcome of a random forest depends on the results of each decision tree. A major advantage of the random forest approach is that it can handle large datasets, due to its robustness to noise [11]. The steps of the learning process normally consist of problem identification and data collection, and then data preprocessing, which are followed by training the model using various algorithms. Based on the result, the parameters can be adjusted to retest the model, until the best model is selected. Ideally, the model should then be validated before entering into general use, by testing it on a new set of test data [12].

### Unsupervised learning

In this form of machine learning, the data provided are unlabeled, and therefore the model cannot make any predictions. In turn, the model can look for patterns or abnormalities (e.g., groups of patients who show similar behavior patterns). Often-used algorithms include clustering and principal component analysis [13]. Clustering can be achieved using various algorithms, with k-means clustering and hierarchical clustering used in particular. In the k-means clustering approach, k stands for the number of clusters we wish to define. From the dataset, a k number of data points are selected. The remaining data points are added to the k clusters based on the proximity – they get added to the nearest cluster. Next, the mean of a cluster is calculated, and each data point gets added to the nearest cluster, based on the mean value. This process is repeated with various values of k, until the sufficient ratio of decreased variance inside a cluster and the number of k is achieved. Hierarchical clustering is a process of joining data points into clusters sequentially, until there is one large group. This works on the basis of similarity and distances between data points and can be graphically represented in the form of a dendrogram. We can observe how many clusters (comprised of two data points) there are, and the clustering order –clusters identified last have less similarity to the other clusters. In principal component analysis, the goal is to use the principal components to reduce the amount of original dimension *d* to a new combined number of dimension *k*, with minimal loss of feature information. These principal components are used to explain the variance of the dataset; the principal component with the largest variance will best describe the differences between the dataset samples. By focusing on the most relevant features, the complexity of the data and the algorithms can be reduced, thus making models simpler and more robust [10,13].

### Reinforcement learning

This form of machine learning uses a feedback system to find the best possible algorithm. The model interacts with its environment by preforming actions, where each action elicits a response, whether it be positive or negative. Based on the response of the environment, the model will adjust its next action accordingly. The response of the model is therefore determined by the response of the environment and *vice versa* [14].

Compared with classical statistics, machine learning provides multiple advantages that can complement, or even surpass, findings that are revealed by pure statistics. As opposed to classical statistical approaches, machine learning allows for predictions, while enabling the integration of clinical, biological, and epidemiological data [15]. Classical statistical approaches are designed in a way that allows only a small number of variables to be analyzed at the same time, which can lead to over-simplification. This might be a problem when dealing with “wide data,” where the number of observations is smaller than the number of features (e.g., genome-wide next-generation sequencing of a small number of subjects can produce millions of data points for each single subject), which is also known as a “high dimensional data problem” [12,16]. Similarly, multiple testing can greatly increase the occurrence of false-positive results, which are also known as type I errors. Type I errors can be minimized by using p-value corrections, although such corrections can, in turn, mask significant differences for a smaller effect, and this can result in false-negative results, which are also known as Type II errors [17]. This is especially dangerous when studying multifactorial complex disease states, such as suicidal behavior, where a combination of often small impact risk factors might enable manifestation of the state.

For those interested in more details on machine learning applications and its practical use, a good understanding of mathematics, statistics, and computing is needed. Multiple literature sources are available, such as Bishop [9] and Hastie et al. [18], as also for software such as Python (e.g.,Scikit-learn) and R (e.g.,caret).

## USE OF MACHINE LEARNING IN PSYCHIATRY

Machine learning has made significant progress from its beginnings in the 1950s, when Samuel designed a simple program that could play a game of checkers [19]. In recent years, machine learning has become a promising tool, and various real-life applications already use its advantages, for tasks such as email spam filtering, weather and traffic prediction, and image and speech recognition [20]. Machine learning has also been recognized as a novel emerging technology in biomedical research [21-23], including psychiatry [24]. For example, a recent study by Qi et al. (2020) used machine learning algorithms to identify micro-RNAs expressed in the blood of patients suffering from major depressive disorder that differed significantly from micro-RNAs expressed in the blood of healthy control subjects [25].

Two of the phenomena within psychiatry that would gain significantly from big data analysis are suicidal ideation and suicidal behavior (including suicide attempts and completed suicides). In recent years, progress has been made in patient stratification based on their clinical data and health records, which should help in the implementation of machine learning approaches. Indeed, machine learning approaches have already been used for analysis of social-media data [26-28], health records and questionnaires [29,30] and suicide notes [31-33].

These studies have highlighted the potential of machine learning and its use in the social aspects of suicidality research. As these data are often self-reported, there is the potential for multiple sources of bias. The process of assessing suicide risk is a difficult and complicated task that rests heavily on the shoulders of healthcare providers [34]. The classical approach of diagnosis that consists of symptom-listing criteria would benefit greatly from the use of biomarkers [35].

People with suicidal ideation and behavior are often in contact with primary care or emergency department employees, who do not always have the time or training to be able to identify the needs of people with suicidal tendencies [36,37]. Studies have shown that up to 80% of inpatient suicide victims deny suicidal ideation in their last communications with a clinician [38], which further highlights the need for the use of biology-based biomarkers. Based on accumulated studies on families, twins, and adoptees, it has become clear that as well as environmental factors, genetics also account for more than 40% of the variability in suicidal behavior [39]. Multiple studies have examined potential biomarkers of suicidal behavior to date. However, despite this large body of work to address the biological components of suicidal behavior, little progress has been made in the identification of specific and precise biomarkers [40]. This might be due to the complexity of suicidal behavior and the data acquired from suicide research. Focusing on an interconnected network of various biomarkers, genetic influences, and environmental factors, while using the advances of both classical statistical and machine learning approaches, might therefore pave the way to a new era of understanding of suicidal ideation and behavior.

## MACHINE LEARNING AND HIGH-THROUGHPUT DATA OF BIOLOGICAL COMPONENTS IN SUICIDAL BEHAVIOR

In suicide research, single biomarker associations such as particular single-nucleotide polymorphisms (SNPs) and mRNA transcripts or metabolites have shown some degree of repeatability between different studies, although this has been limited. Therefore, inconsistencies between studies are more or less present at all times. To overcome small-scale analysis, meta-analyses that combine and reanalyze all of the available data on particular biomarkers and phenotypes have appeared frequently. The more precise estimates of the effect sizes and the markedly increased statistical power are the most important advantages of such meta-analyses. Furthermore, their particular value is the production of consistent results from inconsistent results of individual studies, which thus gives meta-analyses a clinical value [41].

In suicide research, one interesting example is the serotonin transporter gene, which has been widely studied as one of the most important genes in serotonin signaling that is believed to be significantly disrupted in suicide. Data on the polymorphism of “serotonin-transporter-linked polymorphic region (HTTLPR) 5” that is located upstream of the transcription start site can be followed through the evolution of meta-analyses [42-46]. This started with the first in 2003, which included 12 case-control studies [47]. At that time, significant associations with suicidal behavior were defined. Five more meta-analyses followed, with each presenting somewhat different results [42-46]. The last meta-analysis was performed in 2019 [48], and the number of studies had then increased from the 12 of the initial meta-analysis to 45, with an increase from roughly 2500 subjects to 15,000 subjects, respectively. The main result of this final meta-analysis was an association between the low expressing allele and violent suicide attempts, which has often been apparent in such data. On the other hand, no associations with any other suicide phenotypes were apparent, indicating a very particular role of the serotonin transporter in suicidal behavior.

As suicidal behavior, or the suicidal phenotype, cannot be explained by variations in a single marker, it appears instead to be the result of numerous variations with small effect sizes across a broad network of markers. This has thus oriented further research toward the “-omic” approaches. Staying at the level of DNA, we need to consider the various genome-wide association studies (GWAS) where several hundred thousand polymorphisms have been interrogated at once, and together with environmental factors, these have been shown to contribute to the particular phenotype. In a comprehensive review, Gonzalez-Castroet al. [49] used a computational systems biology approach to analyze the available data from GWAS on suicidal behavior. The gene ontology analysis gave seven statistically significant results: Regulation of glucose import in response to insulin stimulus; regulation of protein localization to the cell membrane; positive regulation of endopeptidase activity; heterotypic intercellular adhesion; regulation of myocardial contraction; positive regulation of protein localization to the cell membrane; and positive regulation of protein localization to the cell periphery. In addition, analysis according to the Kyoto encyclopedias of genes and genomes (KEGG) biological pathways gave three statistically significant results: Aldosterone synthesis and secretion, the Rap1 signaling pathway, and arrhythmogenic right ventricular cardiomyopathy. However, for the KEGG analysis, all of these might be linked to suicidal behavior to some extent only through other comorbidities, psychiatric disorders, and stress, among others, which could, of course, all represent predisposing factors for suicide [49].

Similar approaches have been used with other high-throughput data, such as epigenome-wide association studies (EWAS), where again several hundred thousand markers were analyzed, in this case in terms of DNA methylation of CpG dinucleotides. In a recent study by Fiori and Turecki [50], 11 such studies were listed. This EWAS approach appears to be promising for determination of the relationships between differentially methylated regions (DMS) belonging to distinct biological pathways, and also in an understanding of the interactions between methylation of a specific locus and its nearby genomic regions. However, the main disadvantage of EWAS has been the lack of significant overlap between the candidate gene approach and EWAS [50], which is very similar to the weaknesses determined from the GWAS [49].

This lack of overlap between hypothesis-free driven approaches and candidate gene studies, and the uncertainties associated with novel genes in terms of their biological relevance and relatively weak statistical significance, might be what provoked further analyses like that of Sokolowski and Wasserman [51]. In their study, they searched through studies involving candidate SNPs, GWAS, copy number variations, linkage, whole-exome sequencing, mRNAs, proteins, and micro-RNAs to build a synopsis of 106 genes that were associated with suicidal behavior. Their study was indeed comprehensive and extended the interest in the spatial-temporal development of the human brain. Furthermore, it represented a trigger to ask why artificial intelligence has not been used more often in investigations into the discovery of suicidal behavior biomarkers. The amounts of data available are huge, and the search for particular biomarker patterns in suicidal behavior might just benefit from such machine learning approaches.

## GENE AND GENOME-BASED STUDIES OF SUICIDAL BEHAVIOR AND MACHINE LEARNING

Although GWAS are relatively “popular” due to their affordability and the genome-wide nature of the biomarker cover, the data are only rarely analyzed through machine learning. Ruderfer et al. (2019) showed the heritability component of suicide attempts, and also its complex genetic nature, with partial, although at the same time distinct, overlap with psychiatric disorders. Population samples from both the UK Biobank and Vanderbilt University Medical Center (VUMC) were genotyped using microarrays, and the predicted probability of attempting suicide was calculated using random forest algorithms. The cohort from the UK Biobank comprised 2433 cases who reported that they had attempted suicide (according to mental health assessment through an online questionnaire), along with 334,766 controls. The clinical features for the VUMC cohort comprised 3250 cases with suicide attempts and almost 3 million patients derived from the electronic healthcare records. The heritability estimates of suicide attempts for both cohorts showed significant genetic correlation and comparable common variation of about 4% for both the UK Biobank (h^2^ SNP = 0.035; p = 7.12 ×10^−4^) and the VUMC (h^2^ SNP = 0.046; p = 1.51 ×10^−2^). Significant correlations were also seen for insomnia and for several psychiatric disorders or traits, such as depressive symptoms, neuroticism, major depressive disorder, and schizophrenia [52].

Discrimination between suicide attempters and non-attempters that were based on a prediction model of SNPs was performed on psychiatric patients by Baca-Garcia et al. (2009). The genotyping of 840 SNPs in 312 different genes associated with brain function and development was carried out for 277 male patients. Using only three SNPs, it was possible to accurately group 67% of the male suicide attempters and non-attempters (i.e., rs10944288 in *HTR1E* [5-hydroxytryptamine receptor 1E], hCV8953491 in *GABRP* [γ-aminobutyric acid type A receptor subunit Pi] and rs707216 in *ACTN2* [actinin a2]).This provided relatively promising future guidelines for genetic tests that would facilitate psychiatrists in their clinical work, where timely and objective identification of suicide attempters is of great importance [53].

A smaller scale study on 225 patients from the European Group for the Study of Resistant Depression (GSRD) and 12 SNPs from candidate genes (i.e., *HTR2A* [5-hydroxytryptamine receptor 2A], *COMT* [catechol-O-methyltransferase], *ST8SIA2* [ST8 α-N-acetyl-neuraminide α-2,8-sialyltransferase 2], *PPP3CC* [protein phosphatase 3 catalytic subunit γ] and *BDNF* [brain-derived neurotrophic factor]) was performed to investigate the interactions between SNPs and clinical variables, including suicidality, in treatment-resistant depression (with Hamilton Rating Scale for depression [HAM-D] >17). Based on machine learning and clustering algorithms, three SNPs (i.e., rs6313 in *HTR2A*, rs7430 in *PPP3CC*, and rs6265 in *BDNF*) and absence of melancholia grouped 62% of the patients in the same group of therapy non-responders (HAM-D<17). Although suicidality was shown to be an independent clinical variable for treatment prognosis in previous GSRD, no particular association was defined for this model [54].

## METABOLITE-ANALYSIS-BASED STUDIES OF SUICIDAL BEHAVIOR AND MACHINE LEARNING

Determination of metabolites through liquid biopsies of venous blood is a relatively noninvasive approach that can provide a wide range of information. A comprehensive metabolome analysis of 123 metabolites on three independent cohorts of psychiatric patients (with major depressive disorder, bipolar disorder) without and with medication has been performed. An important general finding was that metabolic profiles can be used to evaluate the severity of depression, and five metabolites were associated with severity of depression in the three cohorts (i.e., 3-hydroxybutyrate, betaine, citrate, creatinine and γ-aminobutyric acid), independent of medication and diagnosis. Furthermore, several metabolites were also associated with suicidal ideation (i.e., kynurenine pathway metabolites and citrate), which allowed determination of the patients with and without suicidal ideation [55].

In the French Network of Bipolar Expert Centres, 635 bipolar patients were included in a study of emotional hyper-reactivity, with several different biological factors also measured. Using a machine learning algorithm, it was determined that the patients with emotional hyper-reactivity had significantly higher levels of suicide attempts (*p* = 1.4 × 10^-8^), and also systolic and diastolic blood pressure (*p*< 1.0 × 10^−8^) and high-sensitivity C-reactive protein (*p*< 1.0 × 10^−8^). Based on these three measurements, these patients could be designated with emotional hyper-reactivity with 84.9% accuracy [56].

## EPIGENOME-BASED AND TRANSCRIPTOME-BASED STUDIES OF SUICIDAL BEHAVIOR AND MACHINE LEARNING

A recent study by Bhak et al. (2019) examined whether machine learning algorithms can accurately classify subjects based on their DNA methylation and gene expression status in peripheral blood. Altogether, this study included samples from 56 suicide attempters, 39 major depressive disorder patients, and 87 healthy controls. After next-generation sequencing, the significant results were used as model features; i.e.,DMS and differentially expressed genes (DEGs). Three different comparison models were built to classify: Suicide attempters from patients with major depressive disorder (initially using 7353 DMS, no DEGs); patients with major depressive disorder from healthy controls (initially using 12633 DMS, 16 DEGs); and suicide attempters from healthy controls (initially using 10412 DMS, 154 DEGs). After this testing, the model features decreased, respectively, to: 69 DMS; 80 DMS; and 95 DMS plus 7 DEGs. All three of these models managed to classify patients and healthy controls with good accuracy (>86%). Additional psychiatric score regression was developed using the HAM-D17 and the Beck scale for suicidal ideation questionnaires, where DMS and DEGs correlated with the results of the questionnaire, initially using 2150 DMS plus 80 DEGs for correlation with HAM17, and 1273 DMS plus 82 DEGs for correlation with suicidal ideation. Between the two regression models, 139 markers overlapped. Similarly, model testing then resulted in decreased numbers of features for HAM17 (810 DMS plus 48 DEGs) and suicidal ideation (467 DMS plus 51 DEGs). Finally, gene ontology enrichment was carried out, but no significant enrichment was observed between suicide attempters and patients with major depressive disorder. However, there was enrichment in the Hippo signaling pathway in patients with major depressive disorder, compared to healthy controls. The Hippo signaling pathway is named after the Hippo protein kinase, and it represents a key pathway in animal development and growth, as it regulates the size of the organs. This pathway is also involved in antidepressant responses, although as the majority of these patients with major depressive disorder were prescribed with antidepressants, this result needs to be further investigated. For the regression models, the cell-adhesion protocadherin gene family was enriched in suicide attempters compared to the healthy controls for both of the regression models [57].

## IMAGING STUDIES OF SUICIDAL BEHAVIOR AND MACHINE LEARNING

Imaging technologies have been widely used in psychiatric disorders [58]. There have been four studies to date that have used various imaging techniques to generate machine learning models that can differentiate between groups of subjects.

Just et al. (2017) examined 17 suicide ideators and 17 control subjects, and searched for functional magnetic resonance imaging (fMRI) alterations in neural signatures and the detection of emotional components in these neural signatures. Words can evoke emotions, which result in brain activity; the premise behind this study was therefore that the members of each group will differ in their neurocognition patterns. While measuring brain activity, those in each group were presented with stimuli according to 30 concepts that were divided into the categories of suicide-related and positive and negative concepts, with each presented for 3 s. As well as the location and intensity of the responses, the data were compared with data from known emotional states, with the specific emotional components analyzed as *Sadness*, *Shame*, *Anger*, and *Pride*. Using a Gaussian naïve Bayes machine learning algorithm, a model was designed. This provided distinction between the groups with 91% accuracy on the basis of the six concepts of “*death*”, “*cruelty*”, “*trouble*”, “*carefree*”, “*good*”, and “*praise*” and the five regions of the superior medial frontal area, the medial frontal/anterior cingulate, the middle temporal, the inferior parietal, and the inferior frontal. In terms of the concepts, “*death*” was the greatest discriminator between the groups. In terms of the specific emotional components, for the suicide ideation group, “*death*” evoked more *Shame*, “*trouble*” evoked more *Sadness* and less *Anger*, and “*carefree*” evoked less *Pride*. The biggest difference in the brain regions involved were seen for the anterior frontal region, where a stable signal was detected only for the control group, and the inferior parietal region, where the signal was more stable for the suicide ideators. Furthermore, of the 17 subjects in the suicide ideation group, nine had undergone a previous suicide attempt. Here, the machine learning model differentiated between these two subgroups of the suicide ideators with 94% accuracy. The concepts that best discriminated between the subgroups of suicide ideators were “*death*”, “*lifeless*”, and “*carefree*” with differentiation of the response seen in the superior medial frontal area, the medial frontal/anterior cingulate, and the middle temporal. For the specific emotional components in the subgroup with suicide attempts, “*death*” evoked less *Sadness*, while “*lifeless*” evoked more *Anger*. They then considered the fMRI data of 21 additional suicide ideators who had originally been excluded from the study due to the lower quality of their measurements. These further suicidal ideators were tested against the 17 subjects in the original control group using the machine learning algorithm model. Although the accuracy of the model decreased to 87%, this still represents accurate classification. When they then compared these further 21 suicidal ideators with the suicidal ideators subgroup without previous suicide attempts (i.e., N = 8), the accuracy further decreased to 61%. This indicates that when a model is based on quality data, it can potentially work sufficiently well on noisier data, while the reverse is not true. Altogether, this study provides an insight into the alterations in the way people internalize different concepts, and how this can affect their perspective and psychosocial state [59].

Resting-state functional connectivity (rs-fc)MRI is an imaging technique that is used to determine spatially separated brain regions that are activated at the same time, with the postulation that such joint activation represents an association. This method was used by Caceda et al. (2018) to investigate acute suicidal behavior not as a personality trait, but as a state. To do so, they compared rs-fcMRI of 10 recent suicide attempters (<3 days after their suicide attempt), nine suicide ideators, 17 depressed nonsuicidal patients, and 18 healthy controls. The rs-fcMRI data were analyzed using support vector machine data-driven neural pattern classification. As the sample sizes were small, instead of the previously described train–test dataset approach, they followed the leave-one-outcross-validation that is based on prediction of the status of a single subject based on the values of all of the other subjects. Twenty-one spatially independent components were compared. This model successfully separated the recent suicide attempters from the suicide ideators with 78% accuracy. In these recent suicide attempters, both positive (default mode network: Limbic) and negative (default mode network: Insula) functional connectivity was observed. To further asses the model, the recent suicide attempters were imaged again after 5-7 days (although only seven agreed to participate). This additional study, however, did not confirm the previous results. For the comparison of the re-imaging of these now deemed stable suicide attempters and the suicidal ideators, the mean accuracy decreased to 58%. Similarly, the statistical significance of the model prediction dropped for the comparison of these recent suicide attempters with all of the depressed subjects (mean accuracy, 53%), and the comparison of the subjects who had shown suicidal behavior anytime during their life with those without any suicidal behavior (mean accuracy, 54%). While the numbers of samples were low here, this study still provides an insightful view into the poorly understood acute suicidal behavior [60].

Gosnell et al. (2019) used both rs-fMRI and rs-fcMRI with 63 suicidal and 65 nonsuicidal psychiatric inpatients to measure their structural and resting-state functional connectivities. They designed a random forest classification model that used 316 structural features and 8256 resting-state functional connectivity features, which were reduced to 7 and 40 features, respectively, for the final model. Here, all 47 of these features were significantly different between the two study groups of suicidal and nonsuicidal psychiatric inpatients. Signals from various brain regions were involved, which included the frontal and middle temporal and other brain regions (e.g., amygdala). Using this final model, the separation of these study subjects was predicted with 79% sensitivity. This final model was later tested on a new set of subjects (i.e., an independent sample; n = 32), with a sensitivity of 81%. Six resting-state functional connectivity features were present in the independent sample group as well, with three in the frontal region where changes in resting-state functional connectivity features have been associated with suicidal behavior before. Changes in connectivity between the studied groups were observed for the frontal middle and superior gyrus, which showed altered connectivity with regions such as the rolandic operculum, insula, and putamen (all of which are involved in the reward circuitry). The amygdala and middle temporal pole had decreased resting-state functional connectivity, indicating potential dysregulation of emotion. These results therefore indicate that changes in structural connectivity are associated with suicidal behavior [61].

Recently, Weng et al. (2020) used machine learning of data based on structural MRI from 41 depressive patients with suicidal ideation, 54 depressive patients without suicidal ideation, and 58 healthy controls. They designed multiple models using several machine learning algorithms to compare the depressive patients who showed suicidal ideation with those without suicidal ideation, as a combined group of depressive patients without suicidal ideation and healthy controls. Later they separated this combined control group into two subgroups to increase the balance proportion (where both subgroups contained depressive patients without suicidal ideation as well as healthy controls), and they use these groups for the training and testing of the model. The model with the best prediction classified the subjects with 85% prediction accuracy. Compared to the imaging methods of the two previously mentioned studies, structural MRI takes less time and has lower technique requirements, making it an easier method to use [62].

Imaging studies coupled with machine learning algorithms can help to identify new neuroimaging markers, which might enable stratification based on patient suicide risk, and help in the design of the best therapies and treatment opportunities for individual patients. Such studies can help in the identification of brain regions that might represent targets for transcranial magnetic stimulation (which is already in use for treatment of depression), while the responses to the concepts can be used in psychotherapy approaches.

## CHALLENGES AND OPPORTUNITIES IN THE USE OF MACHINE LEARNING IN HEALTHCARE

Looking at the publication history, an exponential growth in the number of publications related to healthcare can be seen, including for psychiatry. However, as with other young and expanding fields, multiple challenges and questions continue to arise, for which we do not have the complete answers yet.

As observed in the studies using machine learning algo­rithms for suicide research described above (detailed information in Table 1), standardization is needed in terms of data presentation and the description of the model characteristics. As different models use different types of machine learning and algorithms, direct comparisons are not really possible. Indeed, the above-mentioned studies used different measurements for model efficiency, which ranged from accuracy, specificity, sensitivity, area under the receiver operating characteristics curve (AUC), to the receiver operating characteristic curve (ROC). The number of studies that have focused on the biology has also been low and models have often not been validated with external samples, which leaves the need for additional replication studies [63].

**TABLE 1:**
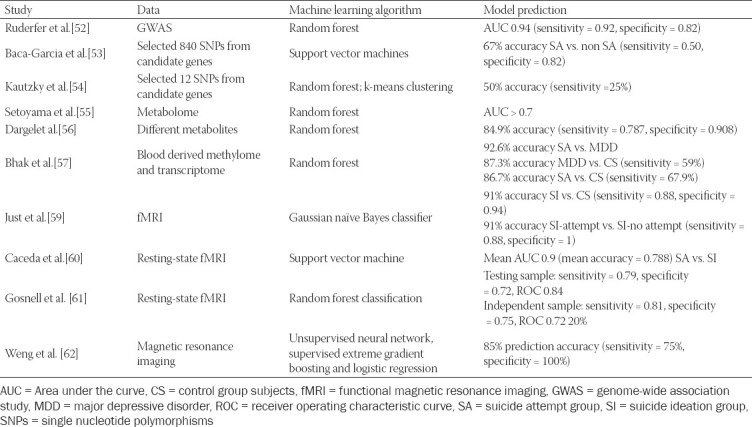
Studies of suicidal behavior that have included machine learning algorithms and models

Machine learning is comprised of many steps where human intervention can impose additional bias on the data collection, such as racial and gender differences [64], and study samples not reflecting the general population [65]. In addition, the models aim to predict whether a person is at risk for suicidal behavior, while they fail to determine the time component of this risk. Such predictions open the question whether a life-time threat of suicidal behavior exists, and the ethical considerations behind such powerful statements, not forgetting their effects on the person concerned.

Once validated and robust models are available, the goal will be to use these not only for strictly research purposes but also to provide healthcare services as well. Both healthcare and research personnel would thus benefit greatly from machine-learning-oriented training [65]. One of the important steps might therefore be data reduction, which is usually applied during model testing state, where as much data as possible are removed without losing or changing the conclusions obtained from the model. This enables the model to be more easily interpreted and to be available to a broader healthcare and research community [12]. Finally, the question of ethics needs to be addressed, in terms of the protection of privacy, data, and patient information.

## CONCLUSION

Machine learning is a young and rapidly evolving research field that allows the handling of complex high dimensional data that can aid in the prognosis, diagnosis, treatment, and clinical workflow for various disease states, including mental disorders [66]. In addition, machine learning approaches are cost effective and noninvasive, which means that it represents a potential complementary tool for psychiatrists and other researchers and healthcare providers.

As seen from the study of Caceda et al. [60], when it comes to risk determination for suicidal behavior, time is of the essence. Once specific and sensitive machine learning models have been constructed and validated in independent sets of patients, the time component of machine learning will greatly depend on the clinical infrastructure, computational power, and skilled personnel. There is, however, probably a long way to go before machine learning can be implemented and used routinely in the clinical setting. Large amounts of unbiased data will be needed, together with the information technology infrastructure, and also adequate regulations according to law as related to institutions such as medicine agencies and health insurance companies.

Despite the numerous challenges that await, machine learning approaches hold a promise for better detection and understanding of suicidal ideation and behavior. When focused on the data that can be obtained from people with suicidal ideation or who show suicidal behavior, or even from those who have died by suicide, we believe that machine learning will allow us to determine the important networks of molecular biomarkers that can be traced as part of the diagnosis, treatment, and monitoring of psychiatric patients, to enable a new era of precision and personalized medicine.
